# Applying Laws Across Time: Disentangling the ‘Always Speaking’ Principles

**DOI:** 10.1093/ojls/gqae014

**Published:** 2024-05-10

**Authors:** Martin David Kelly

**Affiliations:** School of Law, University of Edinburgh

**Keywords:** legislation, law, constitution, legal interpretation, originalism, adjudication

## Abstract

Common-law judges frequently claim to apply the ‘always speaking’ principle. But they recognise that they are not clear on what it means, with Lord Leggatt recently calling the metaphor ‘enigmatic’. In this article, I seek to clarify this by showing that the ‘always speaking’ metaphor is associated with at least *four* different types of principle, each of which responds to a distinct issue (although there is a common theme: change over time). I explore the origins of the ‘always speaking’ metaphor, distinguish the four issues and explain how they relate. I argue that it is important to disentangle the four types of ‘always speaking’ principle, with a focus on distinguishing principles of dynamic (versus originalist) interpretation from principles that empower judges to strain or ‘recast’ legislation to deal with new developments sensibly. In doing so, I analyse and critique the judgments in the recent UK Supreme Court case of *News Corp*.

## 1. Introduction

For Lord Steyn, it is of ‘constitutional significance’ that legislation is ‘always speaking’.^1^ But many lawyers have never even heard of this metaphor, and it is often misunderstood. The phrase ‘always speaking’ appears in decisions across the common-law world, including in no fewer than six UK Supreme Court decisions between March 2020 and April 2021.^2^ Lord Burrows has claimed that, although it is ‘trite law that, at least in general, a statute is “always speaking” … what this precisely means is open to debate’; and Lord Leggatt agreed, in the recent *News Corp* case, adding that the metaphor is ‘arresting but enigmatic’.^3^

In this article, I argue that the ‘always speaking’ metaphor seems enigmatic because it is associated with at least *four* different types of legal principle.^4^ Each addresses a distinct issue, albeit with a common thread: the relation between legislation and *change over time*. These four issues arise for utterances—ie uses of language (whether oral or written)—generally, not just for *legal* utterances, and so I frame them here in general terms:

1. **Operation** issue: at which time(s) is an utterance operative?
2. **Meaning** issue: by reference to which time(s) is the meaning of an utterance determined?
3. **Novelty** issue: does an utterance apply to things not known/foreseen when it was made?
4. **Implicit modification** issue: does an utterance implicitly modify an earlier utterance?

The conflation of these issues has led to flawed decisions in numerous cases (across many jurisdictions), with judges giving the wrong reasons to justify their decision and/or arriving at an incorrect decision.^5^ In this article, I highlight the key differences between these issues and show why they make it problematic to label our responses to all of them ‘the “always speaking” principle’. I then illustrate the problem by analysing the judgments in *News Corp*, criticising the leading judgment of Lords Hamblen and Burrows for conflating these issues and praising Lord Leggatt’s concurring judgment. I begin with the *operation* issue, which is the one that the metaphor was coined to address and the only one for which it makes sense.

## 2. The Operation Issue

### A. Types of Utterance

Many of our utterances are intended to apply to the world; so, to assess whether they apply to a given set of facts, we need to know the time(s) at which they are operative. Some utterances are directed only to the facts at the moment when the utterance was made. If I say that ‘the sky is beautiful’, then I claim only that it is beautiful *at that specific time* (maybe it was beautiful for only a fleeting moment). Let us call such utterances ‘momentary’. Other utterances are intended to be operative *across a period* of time. If I say to my students ‘You may ask me questions before your exam’, then this is to apply for an *extended* time: from that point onwards, until the exam commences. Let us call these ‘extended’ utterances. And if I advise my students to ‘always treat people with respect’, then this is to be operative on an *ongoing* basis, indefinitely. It is this type of utterance that we call ‘always speaking’. I signalled this linguistically—by using the word ‘always’—but I could instead have said ‘never treat anyone with disrespect’, or even just said ‘be respectful to people’ (and thereby left the students to infer, from the context, that my advice is to be treated as ongoing).

Other utterances have a more complex relationship to time. To say that students who fail the exam must do the resit, I should (strictly) say: ‘if you shall have failed the exam then you shall be required to do the resit’. Using the future tense (‘shall be’) indicates that the resit (at t_3_) is *after* my utterance (at t_1_); and using the future perfect tense (‘shall have’) indicates that failing the exam (t_2_) comes *after* my utterance (at t_1_) but *before* the resit (at t_3_). But we need not follow these grammatical rules strictly. The order of these events in time is obvious, so I can just say ‘if you fail the exam then you must do the resit’—using the present tense for both verbs (‘fail’ and ‘do’)—and let the context do the temporal sorting.

### B. Present-Tense Drafting

The ‘always speaking’ metaphor was pioneered by George Coode, in the 1840s, to show when we can (and should) use the present tense for future events.^6^ Coode noticed that legislative drafters were reluctant to do so, and this was leading to torturously drafted clauses such as:

[I]f a person *shall be* convicted of … and if he *shall have been* before convicted of the same offence; and if he *shall not have* undergone the punishment which he *should have* undergone for the offence of which he *shall have been so before* convicted …^7^

This clause could be written much less awkwardly as:

If a person *is* convicted of … and if he *has been* convicted of the same offence; and if he *has not* undergone the punishment which he should have undergone for the offence of which he *was* convicted …^8^

For Coode, statutes not only can but *should* be written like this: using present (and past) tense drafting makes legislation shorter and more easily understood. But Coode noted that:

It is supposed sometimes that it is necessary to [use] the future or perfect future, for fear that if it were expressed in the present tense, as ‘when any person is aggrieved’, the law would operate only upon cases existing at the moment of the passing of the Act; or that if it were expressed ‘when any person has been convicted’, the law would be retrospective, and apply only to convictions previous to the passing of the Act.^9^

But, for Coode, these fears were ‘entirely founded on a mistake’; and, if ‘the law be regarded while it remains in force as *constantly speaking*, we get a clear and simple rule of expression’: that laws should be drafted to describe ‘facts concurrent with its operation, as if they were present facts, and facts precedent to its operation, as if they were past facts’.^10^ Coode’s approach has been very influential, and laws are now routinely drafted in this way.

Coode did not explain why he thought the phrase ‘always speaking’ is apt to capture this. But consider a ‘No Exit’ sign above a door. Surely, this does not ‘speak’ only to those who happened to be present when it was first installed: it is not a momentary utterance. Instead, it ‘speaks’ to everyone who approaches that door, on an ongoing basis, indefinitely. And, as one may (in theory) seek to exit by that door *at any time*, it needs to be ‘always speaking’. Now, of course, a sign does not literally *speak*.^11^ But imagine that the door is in a building frequented by blind people, and it is decided to install a sound system, next to the door, that will play a recording of someone saying ‘No Exit’. This auditory ‘sign’ would not achieve its purpose if it were to play the recording only *once*. Instead, the recording must be played on a continuous loop: it needs to be *always speaking* the words ‘No Exit’.

This analysis of what we might call the ‘temporal application profiles’ of these different types of utterance helps us to understand the time(s) at which our *laws* are to be operative. For our laws are typically not momentary, and a great many of them are ‘always speaking’.

### C. Laws and the Operation Issue

Laws are usually intended to be operative—and thus to guide our behaviour—on an ongoing basis (or, at least, continuously for an extended time). This is particularly true of *legislation*, as its purpose is typically to create general norms that are to apply to anyone who comes to satisfy (into the indefinite future) a specified description. For example, the offence of handling stolen goods applies not only to those who happened to be handling stolen goods at the moment when it came into force. Instead, it also applies to everyone who *subsequently* handles stolen goods, indefinitely, unless and until repealed. Most legislative provisions are either ‘always speaking’ or are ancillary to an ‘always speaking’ provision.

But statutes are just one kind of legal device that involves ‘always speaking’ utterances. For example, constitutions grant standing permissions (and impose ongoing obligations) that typically operate indefinitely, as do many international treaties. Leases are also often of indefinite duration—whether formally (if terminable only by notice) or in practical terms (eg 999-year leases)—and they contain ongoing permissions, prohibitions (eg as to use) and obligations (to repair etc). Consumer services contracts (eg for broadband or streaming services) are also typically indefinite—operating until they are terminated—and include ongoing obligations (most importantly, to provide the relevant services). And so on.

But not all law-making utterances (nor even all legislative ones) are ‘always speaking’. Some are momentary: their operation/application is exhausted when they are ‘spoken’.^12^ Compare a statute effecting a divorce with one granting a couple permission to marry.^13^ The permission is ‘always speaking’: the couple may marry *at any time* after it comes into force. But divorce is a *one-off* change in legal status: it occurs at a particular moment, and the operative provision then has no further application. If an Act identifies those who are being divorced by using a description, rather than by naming them, then it may be unclear whether it is momentary or ‘always speaking’— as in this tale recounted by Robert Megarry:

[W]hen divorce in the modern sense was possible only by Act of Parliament, an unhappily married Town Clerk was promoting a Waterworks Bill for his town; and in clause 64, mingled with something technical … appeared the innocent little phrase ‘and the Town Clerk’s marriage is hereby dissolved.’ Nobody could explain how the words got there, and, in fact, nobody ever noticed them while the Bill was going through Parliament … [T]he Royal Assent was given, and the town clerk lived happily ever after. At a ripe old age he died, still in harness, and a successor had to be found. The question then arose whether this particular provision was personal to the deceased Town Clerk.^14^

This clause surely applies only to this particular (crafty!) Town Clerk: Parliament used this definite description (‘the Town’s Clerk’) *referentially*—to refer to a particular person—rather than to cover everyone who comes to satisfy that description. So this provision was surely momentary: it would not effect the divorce of every *future* Town Clerk, indefinitely. Its application was exhausted when it came into force: it is not ‘always speaking’.

Other momentary legislation includes Acts that granted amnesty for past offences, eg the Act of Oblivion 1660 (which pardoned crimes committed during the English Civil War) and Acts of Attainder (which convicted a named individual of a specified criminal offence). In these examples, the whole Act (not just the operative provision) might be momentary. This may partly explain why Lord Steyn—in what was, at least until *News Corp*, the leading UK decision on the ‘always speaking’ notion (*Burstow*)—said that ‘*statutes* will generally be found to be of the “always speaking” variety’, instead of statutory *provisions*.^15^ But statutes typically contain both momentary provisions *and* ‘always speaking’ provisions. In fact, most momentary legislative provisions are far more mundane than Acts of Divorce, Oblivion, or Attainder: many simply amend or repeal other legislation. A statutory provision is amended at the moment when the amending legislation comes into force, and the provision that does the amending thereby stops being operative (and similarly with repeal).

These references to legislation coming into force highlight, for law-making utterances, an important distinction between speaking *simpliciter* and speaking *legally/systemically*.^16^ This reflects a distinction—standard in speech act theory—between content and force.^17^ Legislation illustrates this very clearly because, at least in the UK, legislation frequently does not actually come into *force* when it is enacted (when its *content* is first expressed).^18^ The ‘handling’ offence is a good example: it was enacted on 26 July 1968, but it did not come into force until 1 January 1969.^19^ When it was enacted, Parliament ‘spoke’—it said something (ie expressed a content)—but it did not *legally* ‘speak’ (it did not create a norm) until the later time when it came into force.^20^ The ‘handling’ offence became operative only from that time, and it continues to be operative today: it is ‘always speaking’, in the first (and authentic) sense of operating continuously, but only from when it came into force. This content/force distinction will be important when we examine (in section 6) how our four issues relate.

To summarise, it makes sense to use the ‘always speaking’ label for a response to the *operation* issue: we can think of an utterance that is to operate on an ongoing basis as ‘always speaking’ to us, constantly telling us what we are allowed or required to do (or prohibited from doing). And this reflects the origin of the metaphor in Coode’s work on present-tense drafting.^21^

However, from around the late 1960s onwards, the ‘always speaking’ metaphor came to be confused with our other three issues, starting with the meaning issue.

## 3. The Meaning Issue

The meaning issue concerns the time(s) by reference to which the meaning of an utterance is to be determined. There are two main approaches to it. One is that the meaning of an utterance is fixed at the time it was made (ie that it has and retains its *original* meaning); the other is that its meanings are fixed at the times by reference to which it is applied (that it has its *current* meaning).^22^ These approaches are described using various metaphors. The former is often called the ‘historical approach’, the ‘fixed-meaning canon’ or the ‘original meaning rule’ (or just ‘originalism’), and the metaphor here is of the meaning being ‘fixed’, ‘frozen’, ‘ossified’, or ‘static’.^23^ For the latter, the metaphor is of the meaning being ‘mobile’, and it is often called ‘dynamic’ or ‘ambulatory’ interpretation, the ‘living-tree’ approach, or—and here is the problem—‘the “always speaking” principle’.^24^ Indeed, this last phrase is now *most often* used as a label for dynamic (or ambulatory) interpretation.

One of the many pieces of legislation that exemplify a *mobile* meaning is the subject of an important set of cases (the ‘Family Member Cases’) about whether people who stand in particular kinds of relationship count as ‘family’ (for the purposes of tenancy legislation first enacted in 1920). This legislation was designed to prevent a family losing their home when the named tenant dies, by entitling a person who was a ‘member of the tenant’s family’ (and who was living with the tenant at the time of death) to succeed to the tenancy. The courts held that *unmarried* (heterosexual) couples could *not* be ‘family’ (as at 1949), then that they *could* be ‘family’ (as at 1961), and then that *homosexual* couples could be ‘family’ (as at 1994).^25^

The meaning issue—as with the operation issue—often arises for non-legal utterances. A famous example of *static* linguistic meaning is Queen Anne reportedly saying in 1707, of the newly built St Paul’s Cathedral in London, that it is ‘awful, artificial, and amusing’.^26^ The linguistic meaning of those three adjectives has changed radically since 1707: ‘awful’ meant *awe-inspiring*; ‘artificial’ meant *highly artistic*; and ‘amusing’ meant *thought-provoking*. Clearly, Queen Anne’s words must be understood with their meaning at the time she spoke, else we might take her to have criticised the Cathedral (when she was actually praising it). But this was a momentary utterance: it expressed her opinion as at that moment in time, and it was not meant to be operative at any future time (never mind on an ongoing basis).^27^

Contrast my advice to ‘always treat people with respect’: if what counts as *respecting* someone has changed since I gave that advice, then, to comply with it, my students would (at least in theory) need to determine whether they must do what *would have counted—*at the time when I gave this advice—as being respectful, or whether they should, instead, do what *would today* count as being respectful.^28^ I would likely have wanted the content of my advice to reflect changes in the ways that we show each other respect. This is often the case for ‘always speaking’ utterances: when we say something that is to be operative into the indefinite future, it usually makes sense that its content should adjust to reflect the future contexts in which it will fall to be applied. So there is a natural link between ‘always speaking’ utterances and dynamic (or ambulatory) interpretation, and this may partly explain why the latter has come to be labelled ‘the “always speaking” principle’.

But the meaning issue is clearly different from the operation issue.^29^ One key difference is that, whilst it is a *speech act* that can be ‘always speaking’, it is uses of *words* and *phrases* in those speech acts that can have a static or mobile meaning. In a single utterance, I may want my use of one word (‘respect’) to be mobile in meaning but my use of another word (‘treat’) to be static. So the meaning issue is more fine-grained than the operation issue.

An example of an ‘always speaking’ utterance in which a key term should be treated as *static* in meaning is the old adage, ‘serve red wine at room temperature’. This originally meant *serve red wine at around 15–16*°*C* (60°F), which was then the relevant average room temperature (and is also the optimal temperature for serving most red wines). But, at least in the UK, average room temperatures are now approximately 20–21°C (70°F). Thus, many who try to follow this advice are now serving their red wines too warm: ‘room temperature’, in this context, is to be understood by reference to the *original* time—when this ‘always speaking’ utterance was first used—and not the *current* time (ie when we serve the wine). So the mere fact that an utterance is intended to *operate* in future situations (that its application is mobile) does not entail that its *meaning* is to change over time.

Thus, it is important to hold separate two distinct questions: (i) is the utterance ‘always speaking’ (in the authentic sense that it operates on an ongoing basis); and (ii) if so, does it *say the same thing* each time it ‘speaks’ (or, instead, might what it says change over time)? For an utterance to say *something different from* what it originally said, it is *necessary* that it is not momentary (and it will often be ‘always speaking’, in the authentic sense).^30^ But it does not follow that this is *sufficient* for it to be able to change in meaning over time: that would be to confuse a necessary condition with a sufficient condition.^31^ The fact that, in making an ‘always speaking’ utterance, we usually want some of its terms to be understood with a mobile meaning might explain *why* the ‘always speaking’ metaphor has come to be used as a label for dynamic (or ambulatory) interpretation. But this is a mistake nonetheless.

The operation and meaning issues also differ in how controversial they are, and in how the principles that we adopt in response to them may be justified. Presuming that legislation is *operative* into the indefinite future is not controversial: it is usually intended to be ‘always speaking’ in this first (and authentic) sense; and, if not, then the presumption is rebutted.^32^ But how best to address the *meaning* issue can be very controversial. It is most controversial for *constitutions* rather than statutes—particularly in the United States (where ‘originalism’ currently has much academic and judicial support)—but support for originalism is not limited to the United States (or to constitutions).^33^ The current UK position is that legislative *meaning* is presumed to be capable of changing over time; this stance is justified on the basis that the original enacting Parliament intended that its meaning may change, and so the presumption is rebuttable by ascribing to that Parliament a contrary intention.^34^ Therefore, the main justification for *this* type of ‘always speaking’ principle is parliamentary sovereignty. However, responses to the novelty issue may *challenge* parliamentary sovereignty.

## 4. The Novelty Issue

The *novelty* issue concerns how an utterance should apply to something novel, in the sense that it was not known to, understood by, or foreseen by the producer of that utterance.^35^ Since that producer did not, by definition, consider the novelty when they chose their words, it will be (at least partly) arbitrary whether the novelty falls within the content expressed by uttering those words. Often, we will get lucky: this partly arbitrary outcome will be fine. However, in at least some cases, this would generate an unsatisfactory outcome. The ‘wine’ example illustrates this nicely: the change in average room temperatures is novel, and it was presumably not known to, or foreseen by, the original producer of the advice (otherwise they would likely have worded it differently). If we want to follow this advice, what should we do?^36^ Are we to serve our red wine at 70°F, thereby generating unsatisfactory outcomes (ie overly warm red wine)? Or should we ‘recast’ the advice—treat it *as if it were worded differently* (say, as ‘serve red wine at 60°F’)—to generate better outcomes?^37^

In all but very rare contexts, legislation is designed to apply to at least some novelties. For example, it is a crime—in England & Wales—to handle stolen *laptops*, even though laptops did not exist in 1968 (when the ‘handling’ offence was enacted).^38^ No one seriously suggests otherwise, although this point is often confused in the debates over ‘originalism’.^39^ But this is not a complete response to the novelty issue: we still need to determine *which* novelties a legislative provision applies to. According to one approach, the *meaning* should do all the work here, and we should simply let novelties lie where they (linguistically) fall: ‘if things not known or understood at the time an Act came into force fall, on a fair construction, within its words, those things should be held to be included’.^40^ So, as the Act says ‘goods’, and laptops are goods, it follows that handling stolen laptops is an offence. That generates a satisfactory outcome, but we will not always be so lucky: think of what counts (or may, in the future, count) as ‘arms’ for the purposes of the US Constitution’s 2nd Amendment. And the worse the outcome of applying the apparent meaning, the more acute the problem.

This problem is lessened somewhat by the fact that novelties often lead to changes in our language, and thus we can sometimes resolve the novelty issue by applying the *current* meaning. Inventions often lead to linguistic changes: the word ‘laptop’ cannot (by definition) appear in pre-coinage utterances, but inventions also influence our use of *existing* concept terms. For example, *Victor Chandler* (1999) concerned whether Teletext pages (invented in 1970) are a ‘document’ for the purposes of legislation (first enacted in 1952) regulating adverts.^41^ The growth of e-communications has greatly changed how we use the word ‘document’.^42^

However, in many other cases, the legislative meaning has changed since enactment—perhaps even *markedly* so—but not in the ‘right’ way (or not markedly enough).^43^ For example, the Family Member Cases (section 3) involve a change in meaning: as new living arrangements emerged, and as social attitudes towards them altered, the word ‘family’ developed a broader meaning. This enabled a bare majority of the House of Lords to hold, in *Fitzpatrick*, that a long-term cohabiting gay couple were members of each other’s ‘family’. Counsel for Mr Fitzpatrick had implicitly conceded that the *original* (1920) meaning of the legislation did *not* include gay couples, but the majority held that ‘family’ here should be given its *current* (1994) meaning (which, in their view, covered this relationship); and, as the majority considered that this was a satisfactory outcome, they did not, strictly, need to decide whether there was a novelty (and, if so, what that novelty was).^44^

This extending—or repurposing—of our existing concept terms is also evident where the novelty is an advance in our understanding. A good example is *Burstow* (section 2C), which concerned appeals by two defendants—who, by stalking their victims, had caused them to suffer anxiety and depression—against convictions for ‘bodily harm’ offences under an 1861 Act.^45^ The defendants argued that ‘bodily harm’ must be understood as it was in 1861, when *psychiatric* harm was not considered to be ‘bodily’. But the House of Lords unanimously held—in purported application of ‘the “always speaking” rule’—that ‘bodily harm’ (as used in the 1861 Act) should be given its *current* meaning; and, as this included ‘recognizable psychiatric illness’, the Lords upheld the convictions.^46^ I will say more about *Burstow* in section 7B(ii); the key point here is that the novelty—unforeseen advances in medical understanding, especially the connection between mental illness and the brain (as a part of the body)—had, by the 1990s, become reflected in the *meaning* of ‘bodily’ in a way that enabled the Lords to reach an outcome that they considered to be satisfactory by giving the legislation its *current* (1990s)—rather than its *original* (Victorian-era)—meaning.

These examples show a close connection between the meaning and novelty issues, and this may partly explain why the ‘always speaking’ label has come to be used for the novelty issue. But this close connection should not obscure the fact that the two issues are importantly different.^47^ It is true that, in some cases, our response to the meaning issue may happen to neutralise the novelty issue in that case, by (luckily) preventing what would otherwise have been an unsatisfactory outcome. But this is certainly not true of all cases involving novelties.

If the current meaning does not help us, we might reach a better outcome by *straining* the legislative meaning. We usually determine that meaning partly by considering the purposes of the legislation and the consequences of adopting various proposed interpretations of it; and we may be tempted to strain the meaning somewhat, to align our decision more closely with those purposes and/or to generate better consequences. Thus, there is a wide range of possible responses to the novelty issue, depending on just how much straining is permitted. But there are some cases in which the novelty cannot be brought within even a highly strained meaning (whether original or current). The question is then: should we 'recast' the legislation—treat it as if it were worded differently—to generate a better outcome?

A good example is the leading UK case on the novelty issue, *Royal College of Nursing*, which concerned a 1967 Act that partially legalised abortion by creating an exception for ‘when a pregnancy is terminated by a [doctor]’ under specified conditions.^48^ In 1967, abortions were all performed surgically; but, in 1971, a new method (‘medical induction’) was invented and, as it is superior to surgical methods, it quickly became dominant. Medical inductions involved administering the drug prostaglandin intravenously, over 18–30 hours; and, in practice, this was done by nurses (albeit as directed by and supervised by a doctor). Were the nurses acting legally in performing medical inductions?

The question here was *not* whether to give the provision its original or current meaning: no one suggested that its meaning had changed in any material respect since 1967. Nor would straining that meaning suffice to make medical inductions legal: a pregnancy terminated by nurses is simply not one terminated *by* a doctor, just as a portrait painted by an artist working in Rembrandt’s studio—even if it was painted at Rembrandt’s direction, and under his supervision—was not painted *by* Rembrandt. The problem, instead, was that: (i) medical inductions were not contemplated by the 1967 Parliament when it chose which words to enact; (ii) the words it chose simply do not cover them; and (iii) if this novelty was allowed to lie where it fell, then two seriously undesirable consequences would follow. First, abortions would either have to be performed surgically (with a much higher risk to the pregnant woman) or medical inductions would have to be performed by a doctor (which would be a heinous waste of resources), at least until new legislation addressed the novelty differently. Second, the tens (or even hundreds) of thousands of nurses who had performed medical inductions, over a period of almost a decade, had all committed serious criminal offences.

This case generated much judicial disagreement. In the High Court, Woolf J held that the nurses *were* acting lawfully. But the Court of Appeal unanimously reversed that decision: for Brightman LJ, it ‘would be a misuse of language’ to describe these terminations ‘as done “by” a [doctor]’; for Lord Denning MR, they would be legal only if ‘the continuous act of administering prostaglandin [was] done by the doctor personally’; and for Baker J, although their decision may ‘result in a safe and easy method being less used, with consequent hardship or even greater danger to pregnant women’, a judge is not ‘entitled to read an Act differently from what it says simply because he thinks Parliament would have so provided had the situation been envisaged at that time’.^49^ The House of Lords, by a bare majority, reversed the Court of Appeal’s decision. But Lord Wilberforce (dissenting) said of the majority’s opinion that ‘this is not construction: it is rewriting’; and the other dissenter described it as ‘redrafting with a vengeance’.^50^ In our terms, the legislation was *recast* by the majority.

I will say more about this case in section 7B(i). The key point here is that the meaning and novelty issues are importantly different: the former concerns the time by reference to which the meaning of legislation is determined, and the latter concerns how legislation should deal with things or situations that were not known to (or foreseen by) the Parliament that enacted it. Our responses to the meaning and novelty issues are also *justified* differently. Dynamic interpretation is usually justified via parliamentary sovereignty: when we treat legislative meaning as mobile, we do so because we take the enacting Parliament to have instructed us to do so (albeit implicitly). But when judges strain or (especially) recast legislation, this may *challenge* that sovereignty: as it is what the legislature instructed us to do—what it meant by the words that it chose to enact—that is the problem. This raises important *separation of powers* issues. If a court gives legislation its *current* meaning because that is what the original enacting Parliament instructed, this is not only consistent with but *required by* the court’s role as a faithful agent of the legislature. But when a court recasts legislation, it exercises an (interstitial) quasi-legislative power to *change* that legislation. This raises serious concerns about democratically illegitimate and unaccountable judicial power.^51^

The meaning and novelty issues also differ as to the key *rule of law* value of fair notice. The purpose of legislation is usually to guide our conduct; and to achieve that purpose (and for sanctions for non-compliance to be justified), we must have *fair notice* of its content (of what it permits or requires us to do, or prohibits us from doing). This may be achieved under either an ‘original meaning’ or a ‘current meaning’ approach, although fair notice considerations slightly favour the latter.^52^ But if a court recasts legislation to deal with a novelty more satisfactorily, that might be unfair to those who relied on the apparent meaning of that legislation in organising their affairs. Such considerations led Lord Simon to conclude that, ‘in a society living under the rule of law’, those who are subject to the law ‘are entitled to regulate their conduct according to what a statute has said, rather than by … what it would have otherwise said if a newly considered situation had been envisaged’.^53^

This fair notice problem is exacerbated by the lack of guidance on *when* courts will exercise this quasi-legislative power. Evelyn Ellis has claimed—regarding whether courts will read legislation ‘in the way in which they believe Parliament would have wished to phrase it in order to achieve its desired end, had Parliament known of later developments’—that it is ‘well nigh impossible to predict when the judiciary’ will be ‘daring’ enough to treat legislation as if it had been ‘amended’.^54^ This leaves those who seek to comply with a piece of legislation in a quandary: can they rely on the apparent meaning of that legislation, or should they try to predict whether or not a court would be willing to recast it?

A related difference between the meaning and novelty issues concerns the *retrospectivity* of recasting legislation. The power to amend statutes is usually reserved to the legislature not only because it (uniquely) is democratically legitimate and accountable, but also because only the legislature can amend legislation with exclusively *prospective* effect (or with transitional provisions to protect legitimate expectations). But court decisions are almost always fully retrospective in their effect: their main function is to resolve disputes about past events, and if they were not retrospective they would be unable to fulfil that function.^55^

These fundamental issues have led to strident objections to any judicial power to recast legislation: it has been described as ‘unacceptably speculative’ and ‘potentially perilous’.^56^ Judges often—and perhaps understandably—downplay their law-making powers (at least in areas of law that are governed by legislation): they rarely acknowledge that they have recast legislation, and tend instead to present their decisions as being mere *interpretations*. But although there is no bright line that separates (i) adopting a tenable (though strained) interpretation of legislation from (ii) recasting that legislation, it does not follow that there is no difference between them; and there are several clear cases of recasting.^57^

The question of whether judges should strain or recast legislation to address the novelty issue arises only because the legislature has not enacted fresh legislation to deal with the novelty directly. Our fourth issue—*implicit modification*—also concerns how to deal with a novelty, but this time the novelty is itself new legislation (enacted by the same legislature).

## 5. The Implicit Modification Issue

The legislature that enacted a piece of legislation usually has the power, uniquely, to repeal or amend it (by enacting new legislation).^58^ Typically, it does so *explicitly—*by declaring that the existing legislation is repealed (or specifying how it is amended). But sometimes a legislature does not fully address how its new enactment interacts with existing legislation, and so a question may arise as to whether it has modified existing legislation *implicitly*.

This issue is also a *general* one. Returning to our ‘always treat people with respect’ example, imagine that I later tell the same students to ‘always do what you think is right’. Those pieces of advice may come into conflict in some situations, and—if they wish to follow them—my students might need to decide how they interact. I could have modified my first advice *explicitly*, eg by saying ‘always do what you think is right, even if that involves disrespecting someone’. But I did not do this, and so they must decide whether I modified it *implicitly*. This conflict arose because I gave them some *more recent* advice, and we usually treat our later selves as authoritative over our past selves. Hence, it seems natural to resolve the conflict by determining what I meant by my *latest* piece of advice.^59^

A good legal example is the *Oxfordshire* case, which concerned whether 1965 legislation that enacted a startlingly wide definition of ‘town or village green’ had implicitly modified the scope of Victorian-era provisions that also used that phrase (but without defining it).^60^ The question was *not* whether the meaning of these earlier Acts had changed simply through the passage of time. And, while the 1965 Act was a novelty (as it was not known to, or foreseen by, the earlier Parliaments), it arose *from further legislation by the same legislature*. Thus, the question was: what should we take the later (1965) Parliament to have intended, regarding the earlier (1800s) legislation, in enacting those words? So, whereas the novelty issue concerns whether the *old* legislation deals adequately with a novelty (and, if not, whether to strain or recast it), the implicit modification issue turns on the *new* legislation.

There are good reasons for having a general principle that allows implicit modification. Legislative drafters, especially in jurisdictions with large statute books, cannot be expected to identify every provision that their Bill may affect (and explicitly modify them all). This is a counsel of perfection; inevitably, even the most gifted drafter will miss some interactions, and judges should be able to deal with these situations sensibly. As judges can do so by ascribing to the *later* legislature an intention regarding the earlier legislation, this is largely just orthodox statutory interpretation and is thus consistent with parliamentary sovereignty.

However, it is problematic to bring such principles under the ‘always speaking’ label.^61^ This compounds the mistake of extending that label from the meaning issue to the novelty issue (and, of course, it was a mistake to extend it to the meaning issue in the first place). But it also fails to recognise that new legislation, enacted by the same legislature, is a *special kind of* novelty. This has led to several incorrectly reasoned decisions: with judges resolving the implicit modification issue according to the intention of the *earlier* Parliament.^62^

## 6. Relating the Issues

Taking stock, I have highlighted several ways in which these four issues relate to each other. First, they arise for utterances generally (not just for *law-making* utterances, such as statutes). Second, they share a broad theme: the relation between utterances and change over time. Third, if an utterance is ‘always speaking’, in the first and authentic sense of being operative on an ongoing basis (a response to *operation* issue), then it is natural to presume that its meaning may change over time (a response to *meaning* issue); and this may explain why the ‘always speaking’ label has come to be used for a presumption of dynamic interpretation. It also provides a helpful way of thinking about *how* the meaning can change over time: ‘always speaking’ utterances are to be treated as if they are continually being re-uttered; and, as each re-utterance takes place in a new context, it can express a new content.^63^ Fourth, if an utterance is ‘always speaking’ in the first and authentic sense, then we may need to decide how that utterance should apply to a new development (ie a response either to the novelty issue or, if the novelty is new legislation, to the implicit modification issue).

The third and fourth points suggest that legislation must be ‘always speaking’ in the authentic sense—or, at least, that it must not be momentary—for the other issues to arise. It may seem that the *meaning* issue cannot arise for a momentary provision: if legislation applies only to the facts that obtained at the moment it was enacted, then no later change in meaning can alter how it applied. But things are more complex for the other two issues. Although the producer of an utterance can (by definition) become *aware* of the novelty only after making the utterance, the novelty itself may exist at the time of utterance, and so the question of whether to strain or revise the utterance—to deal with the novelty more satisfactorily—might arise. But such situations will be very rare. And the *implicit modification* issue might arise for a momentary provision—a later legislature may amend (or repeal) it implicitly—but, as the application of the provision is already exhausted, it is highly unlikely to arise in practice.

There is a further caveat: there can be a time gap between enacting a momentary provision and it coming into force. The *meaning* may change in that time gap; a *novelty* may arise in it; and the legislation could even be *implicitly modified* before it comes into force. So, strictly, the operation issue is not logically prior to the other three issues. But any time gap between legislation being enacted and it then coming into force is usually quite short, and this complication is thus unlikely to arise very much in practice. Therefore, in the vast majority of cases, the other three issues arise only for provisions that are not momentary. But even if, in practice, for one of the other three issues to arise, it is usually *necessary* that the relevant legislation is operative on an ongoing basis (ie that it is ‘always speaking’ in the first and authentic sense), that is not nearly enough to warrant us to use the label ‘the “always speaking” principle’ for our responses to any of these other three issues.^64^

The meaning issue is, however, logically prior to the novelty issue. The question of whether legislation deals with a novelty satisfactorily can be answered only after identifying its meaning; and, at least in theory, that involves deciding between the original and current meaning.

To summarise: issues 1–3 are, in all but rare cases, sequential. First, we resolve the operation issue; then (if needed) we resolve the meaning issue; and then (if needed) we resolve the novelty issue. This provides us with a decision procedure for novelty issue cases, which I will now illustrate using the recent UK Supreme Court decision in *News Corp*.

This concerned whether legislation (first enacted in 1972) conferring VAT zero-rating on supplies of ‘newspapers’ applied to digital editions of newspapers. The Supreme Court held (unanimously) that it did not. This clearly involved a novelty: digital editions were not known to, or foreseen by, the 1972 Parliament that first enacted this valuable tax status. But, under our decision procedure, we should start by asking whether the legislation is ‘always speaking’ in the first and authentic sense: was it *operative* on an ongoing basis? Clearly, it was: just as VAT was generally to be levied on supplies of goods and services on an ongoing basis, so zero-rating was to apply to such supplies (if they met specified criteria). Next, we identify the *meaning* of the legislation—including, at least in theory, whether it should be given its original (1970s) or its ‘current’ meaning. Once we have sufficiently identified the legislative meaning, we can then assess whether it would generate a sensible outcome for the *novelty* (and, if not, whether we should strain or recast it to generate a better outcome).

The justices in *News Corp* may have benefited from following this decision procedure. The leading judgment—jointly given by Lords Hamblen and Burrows—discussed the ‘always speaking’ principle, but it is not fully clear about which of our issues it responds to. Lord Leggatt’s concurrence, on the other hand, is the best judicial analysis of the ‘always speaking’ notion to date: he recognised the origins of the ‘always speaking’ metaphor (in Coode’s work on present-tense drafting) and correctly distinguished three of our four uses of the ‘always speaking’ label. But Lords Hamblen and Burrows did not directly engage with Lord Leggatt’s analysis, and they score considerably less well on these points.

## 7. News Corp: the Hamblen–Burrows Judgment

### A. Defining the ‘Always Speaking’ Principle?

Lords Hamblen and Burrows framed the case as a battle between: (i) ‘the “always speaking” principle (of domestic statutory interpretation)—which may be thought to support a wide interpretation’; and (ii) ‘the EU law requirement to interpret exemptions from VAT strictly and to give effect to a “standstill” provision’.^65^ But they did not even consider whether the relevant legislation was ‘always speaking’ *qua* ongoing operation. Instead, they recounted the argument of counsel for News Corp that this is ‘a classic example of where an “always speaking” interpretation is appropriate’ because it concerned ‘a technological development’ where the ‘underlying purpose [of] VAT zero-rating carries through to the new development’ (Lords Hamblen and Burrows considered that this was ‘an attractive submission not least because of its rational simplicity’).^66^ This presents the case as turning on the *novelty* issue. Lords Hamblen and Burrows then defined ‘the always speaking principle’ as follows:

[A]s general rule, a statute should be interpreted taking into account changes that have occurred since the statute was enacted. Those changes may include, for example, technological developments, changes in scientific understanding, changes in social attitudes and changes in the law … Exceptionally, the always speaking principle will not be applied where it is clear, from the words used in the light of their context and purpose, that the provision is tied to an historic or frozen interpretation.^67^

Which of our four issues were Lords Hamblen and Burrows articulating a response to here? Clearly not the *operation* issue: they were not saying that statutes are generally operative on an ongoing basis. But this passage seems broad enough to encompass the other three issues: changes in *meaning*, a *novelty* emerging and new legislation being enacted (as one type of ‘change in the law’) which might *implicitly modify* existing legislation. Curiously, Lords Hamblen and Burrows did not explicitly mention changes in meaning. But their claim is a general one, their list of specific types of change is expressly illustrative only, and Lord Burrows had (in his Hamlyn lectures) strongly associated the ‘always speaking’ metaphor with changes in meaning: ‘It is trite law that, at least in general, a statute is “always speaking” or, as it has otherwise been expressed, has an “ambulatory *meaning*”’.^68^

Although it covers the meaning issue, Lords Hamblen and Burrows’s definition mainly presents the ‘always speaking’ principle as being about *novelties*. This is shown by their claim that its ‘great merit’ is that ‘it operates to prevent statutes becoming outdated’ given that it ‘would be unrealistic for Parliament to try to keep most statutes up to date by continually passing amendments to cope with subsequent change’.^69^ This claim makes sense only for a response to the *novelty* issue; regarding the *meaning* issue, it would be absurd to enact new legislation to ensure that statutes which use terms like ‘bodily harm’, ‘member of the family’ and ‘reasonably’ are kept ‘up to date’. How would Parliament even do that, except by constantly re-enacting that legislation using exactly the same words?

### B. The ‘Leading Cases on the Always Speaking Principle’

Lords Hamblen and Burrows did not disclose the origins of their definition, but they then reviewed five of ‘the leading cases on the always speaking principle’.^70^ It is evident, from their selection of these cases, that they have conflated the meaning and novelty issues: three of the cases concern the novelty issue, but the other two are meaning issue cases.

#### (i) Royal College of Nursing

I discussed the first of their cases, *Royal College of Nursing*, in section 4: it concerned the legality of abortions performed by medical induction. The tests that Lords Hamblen and Burrows ultimately applied in *News Corp* are set out in the Lord Wilberforce’s dissenting opinion:

In interpreting an Act of Parliament it is proper, and indeed necessary, to have regard to the state of affairs existing, and known by Parliament to be existing, at the time. It is a fair presumption that Parliament’s policy or intention is directed to that state of affairs … [W]hen a new state of affairs, or a fresh set of facts bearing on policy, comes into existence, the courts have to consider whether they fall within the Parliamentary intention. They may be held to do so, *if they fall within the same genus of facts* as those to which the expressed policy has been formulated [or] *if there can be detected a clear purpose* in the legislation *which can only be fulfilled if* the extension is made.^71^

Lord Wilberforce’s first test here permits at least some straining of the legislative meaning. The ‘genus’ analogy is vague, but it suggests that fairly specific language (the species part) may be read more broadly, such that it applies to something that is not in the same species (ie does not fall within its meaning) but is similar enough to it to be ‘within the same genus’. How far this permits judges to go in straining the legislative meaning was—perhaps deliberately—left somewhat unclear by Lord Wilberforce.

The second test, by contrast, initially appears unconstrained by the legislative meaning. It requires (i) that there is a clear legislative purpose and (ii) that it can only be fulfilled by extending the legislation to the novelty.^72^ This looks like a strong purposivist approach to interpretation; but that appearance is deceptive. Lord Wilberforce added that how ‘liberally’ this test may be applied ‘must depend upon the nature of the enactment, and the strictness or otherwise of the words in which it has been expressed’, and also that, because abortion is a ‘controversial subject involving moral and social judgments on which opinions strongly differ’, medical induction by nurses ‘should not be sanctioned by judicial decision, but only by Parliament after proper consideration of the implications and necessary safeguards’.^73^ Importantly, Lord Wilberforce also imposed an overriding constraint on these tests:

[T]here is one course which the courts cannot take … they cannot fill gaps; they cannot by asking the question ‘What would Parliament have done in this current case—not being one in contemplation—if the facts had been before it?’ attempt themselves to supply the answer, if the answer is not to be found in the terms of the Act itself.^74^

Thus, for Lord Wilberforce, judges must not respond to the novelty issue by adopting a *counterfactual* approach: they must not seek to determine how the original enacting Parliament *would have* deal with the novelty had they known about it. He then opined that the Abortion Act 1967 ‘is not for “purposive” or “liberal” or “equitable” construction’; and, as medical inductions performed by nurses were ‘beyond the legislature’s fairly expressed authority’, they were unlawful.^75^ Lord Wilberforce’s focus was therefore firmly on the legislative *language*—on ‘the terms of the Act’—and he sought to *limit* how far courts are permitted to stray from what Parliament had meant by the words it had chosen to enact.

For Lords Hamblen and Burrows, these passages from Lord Wilberforce’s opinion stated how ‘the always speaking principle is to be applied’.^76^ But this gloss is doubtful (at best): the phrase ‘always speaking’ does not appear in any of the opinions in *Royal College of Nursing* (and it did not make its debut in a reported UK case until 1997).^77^ They also noted that, although this was a dissenting opinion, the House of Lords ‘subsequently approved’ it in *Quintavalle*—another of their ‘leading cases on the always speaking principle’.^78^ This gives our story a curious twist: *Quintavalle*, as we shall see, is a clear case of recasting (or, in Lord Wilberforce’s terms, ‘rewriting’) legislation. But, before we analyse *Quintavalle*, we should examine the second of Lords Hamblen and Burrows’s leading cases: *Burstow*.

#### (ii) Burstow

I introduced *Burstow* in section 4: it was resolved by giving the phrase ‘bodily harm’ in an 1861 Act its ‘current’ meaning; and thus, in light of our improved understanding of the link between the brain (as part of the body) and the mind, the phrase covered recognised *mental* illnesses. So *Burstow* turned on the meaning issue: there was a novelty (our improved understanding), but this had infused our language such that the case could be resolved through a dynamic interpretation: by giving the key legislative phrase (‘bodily harm’) its current meaning.

But Lord Steyn’s leading opinion did not fully comprehend the ‘always speaking’ notion. He (rightly) acknowledged its origin—in encouraging the use of present-tense drafting in legislation—but he then (wrongly) claimed that this ‘drafting technique … brought about the situation that statutes will generally be found to be of the “always speaking” variety’.^79^ The *converse* of this is true: the ‘drafting technique’ of using the present tense for future situations does not *cause* legislation to be ‘always speaking’; rather, it is because legislation is ‘always speaking’ that we can safely use the present tense for future situations.

Lord Steyn then held that: (i) the crucial issue was whether ‘to apply the current meaning of the statute to present day conditions’ or to apply its ‘historical or original meaning’; (ii) resolving this is ‘a matter of interpretation’; (iii) because ‘statutes are usually intended to operate for many years it would be most inconvenient if courts could never rely … on the current meaning of statutes’; and (iv) the 1861 Act is ‘of the “always speaking” type: [it] must be interpreted in the light of the best current scientific appreciation of the link between the body and psychiatric injury’.^80^ Lord Steyn thus clearly treated the ‘always speaking’ principle as a principle of dynamic interpretation (ie a response to the *meaning* issue). But he then claimed that the *Royal College of Nursing* decision is ‘an example of an “always speaking” construction’, despite the question of static versus dynamic interpretation not being a live one in that case.^81^

#### (iii) Quintavalle

Lord Steyn showed a better understanding of *Royal College of Nursing* in the next of Lords Hamblen and Burrows’s five leading cases on the ‘always speaking’ principle: *Quintavalle*. This concerned a 1990 Act that regulated creating and using human embryos, which it defined as ‘a live human embryo where fertilisation is complete’.^82^ In 1990, a live human embryo could be created only by fertilisation; but then a new method—‘cell nuclear replacement’ (CNR)—enabled them to be created otherwise than by fertilisation. The key question was whether a CNR embryo is an ‘embryo’ for the purposes of the 1990 Act. If so, then creating and using CNR embryos would fall within that Act’s strict regulatory regime. But, if not, then CNR embryos could be created by anyone and used for any purpose—eg ‘placing a human embryo created in this way inside an animal’ or using an embryo ‘to create a human clone’—without any legal limits.^83^ The appellate courts were not willing to countenance these troubling outcomes: the Court of Appeal, and then the House of Lords, unanimously held that a CNR embryo *is* an ‘embryo’ for the purposes of the 1990 Act.

*Quintavalle* clearly did not concern the meaning issue: no one was suggesting that the meaning of the legislative phrase ‘where fertilisation is complete’ had changed since 1990. And no amount of straining of that meaning would bring CNR embryos within its scope: they clearly fell outside its meaning (as they are simply not fertilised). So the real question was whether to *recast* this provision—to treat it as if it were worded differently—to avoid the nightmarish outcome that live human CNR embryos were entirely legally unregulated. But Lord Bingham, giving the leading opinion, said, in a now oft-quoted passage, that:

There is … no inconsistency between the rule that statutory language retains the meaning it had when Parliament used it and the rule that a statute is always speaking. If Parliament, however long ago, passed an Act applicable to dogs, it could not properly be interpreted to apply to cats; but it could properly be held to apply to animals which were not regarded as dogs when the Act was passed but are so regarded now.^84^

Thus, Lord Bingham took the ‘always speaking’ rule to be one of dynamic interpretation.^85^ But he did not mention the phrase ‘always speaking’ again, and it is unclear why he made this comment—especially as he then (rightly) treated this as a novelty issue case. Lord Bingham, noting that courts often ‘grapple with the question whether a modern invention or activity falls within old statutory language’—and emphasising ‘the constitutional imperative that the courts stick to their interpretative role and do not assume the mantle of legislators’—then quoted (and purported to apply) the *Royal College of Nursing* tests.^86^ But Lord Bingham, neglecting Lord Wilberforce’s overriding constraint, held that CNR embryos ‘fall within the same genus of facts’ as fertilised embryos and that, given ‘the clarity of Parliament’s purpose’, the 1990 Act *did* cover CNR embryos.^87^ This was clearly a recasting of the legislation.

Lord Steyn, concurring, took a similar approach to Lord Bingham (with whose opinion he expressly agreed). The ‘critical question’ was ‘whether, in the light of a new scientific development, the Parliamentary intent covers the new state of affairs’; and Lord Steyn then also set out (and purported to apply) Lord Wilberforce’s tests in *Royal College of Nursing*.^88^ But, similarly to Lord Bingham, Lord Steyn did not apply them with full fidelity. For Lord Steyn, this was

a classic case where the new scientific development falls within what Lord Wilberforce called ‘the same genus of facts’ and in any event there is a clear legislative purpose which can only be fulfilled if an extensive interpretation is adopted.^89^

Lord Steyn then canvassed two methods of achieving this extension: the first was to treat the words ‘where fertilisation is complete’ as being ‘merely illustrative of the legislative purpose’, thus making those words ‘otiose’; the second was to ‘imply a phrase’ so that the provision reads ‘a live human embryo where [if it is produced by fertilisation] fertilisation is complete’.^90^ Lord Steyn concluded that, if it was ‘necessary to choose’ between these two methods, he would ‘adopt the former’ because it ‘requires no verbal manipulation’.^91^ But ignoring words is just as much a recasting (or ‘rewriting’) of legislation as implying a phrase into it.

Lord Steyn’s opinion, like Lord Bingham’s, mentioned the ‘always speaking’ metaphor. Endorsing his own leading opinion in *Burstow*, Lord Steyn contrasted the ‘always speaking’ approach with ‘historical’ interpretation (ie that legislation ‘must be construed as if one were interpreting it on the day after it was passed’) and said that, as the 1990 Act is ‘always speaking’, the Act ‘may be construed in the light of contemporary scientific knowledge’.^92^ Thus, like Lord Bingham, Lord Steyn treated the idea that statutes are ‘always speaking’ as a principle of dynamic interpretation. But, unlike Lord Bingham, Lord Steyn rightly noted that dynamic interpretation ‘does not solve the problem’ with the 1990 Act.^93^ Lord Steyn did not say *why* it did not solve the problem, although this is presumably because CNR embryos clearly fell outwith even the *current* legislative meaning.

Therefore, although Lord Bingham and Lord Steyn each discussed the ‘always speaking’ principle in their opinions in *Quintavalle*, they each rightly treated it as a novelty issue case. In *News Corp*, Lords Hamblen and Burrows did not engage specifically with either Lord Bingham’s leading opinion or Lord Steyn’s concurrence, so they might have missed that neither of them purported to decide *Quintavalle* by applying the ‘always speaking’ principle (and that Lord Steyn was explicit that he was *not* deciding the case by applying it).

#### (iv) Owens

The fourth of Lords Hamblen and Burrows’s leading cases concerned the *meaning* issue. *Owens* turned on legislation (first enacted in 1969) that enabled divorce where a person’s behaviour is such that their spouse ‘cannot reasonably be expected to live with’ them.^94^ Unreasonable behaviour in a marriage was certainly not a new development, but the major changes in social attitudes towards women since 1969 were presumably not foreseen by the 1969 Parliament and so they count as a novelty. For example, in 1970 a judge held that a husband was ‘not in any sense culpable’ when he used force against his wife in attempting to have sex with her: a decision that, in 2018, Lord Wilson (in the UK Supreme Court) described as ‘inconceivable’ today.^95^ But, as was the case with *Burstow*, the relevant change had become reflected in our language—our use, in this context, of the word ‘reasonably’—such that the courts could apply our current standards of tolerable marital behaviour just by giving the relevant legislation its current meaning (without needing to strain or recast it). As Munby P put it, in the Court of Appeal, the behaviour of spouses must be judged by:

[T]he standards of the reasonable man or woman on the Clapham omnibus: not the man on the horse-drawn omnibus in Victorian times … not the man or woman on the Routemaster clutching their paper bus ticket in October 1969 when the 1969 Act received Royal Assent, but the man or woman on the Boris bus with their Oyster card in 2017.^96^

So, of the first four of what Lords Hamblen and Burrows took to be the leading cases on the ‘always speaking’ principle, two are *novelty* issue cases and two are *meaning* issue cases. The *operation* issue was not contentious in any of them: the relevant legislation was all clearly ‘always speaking’ in the first and authentic sense (ie operative on an ongoing basis).

#### (v) FII GLO

Their fifth case, *FII GLO*, returns us to the novelty issue; this time, the new development was an unforeseen change in the law. Legislation first enacted in 1939 established a special limitation period for actions for ‘relief from the consequences of a mistake’; before 1998, such actions were possible for mistakes of *fact* only; but then the House of Lords changed the common law, by allowing actions for mistakes of *law*, and held that the special limitation period covers them.^97^ The question, on which the Supreme Court divided four to three, was whether the Lords were correct on this limitation point. The majority and dissenting judgments analysed this in terms of whether the legislation is ‘always speaking’.

Lords Reed and Hodge, for the majority, made some progress in disentangling the ‘always speaking’ principles. They said that this ‘somewhat vague expression’ is ‘commonly used in connection with statutory terms which change in their connotations over time’, and they discussed *Fitzpatrick* and *Burstow* (two meaning issue cases).^98^ But Lords Reed and Hodge then discussed *Royal College of Nursing*, *Victor Chandler* and *Quintavalle* (three novelty issue cases), prefacing this discussion with the comment that:

The ‘always speaking’ principle is also invoked where the question arises whether a statutory expression should be interpreted as including a novel invention or activity which does not naturally fall within its meaning, and was not envisaged at the time of its enactment, but may nevertheless fall within the scope of its original intention.^99^

Lords Reed and Hodge also noted (rightly) that *FII* differs from those three novelty issue cases in that they each concerned a novelty that fell *outwith* the legislative meaning (and so the question was whether to *extend* the legislation to include it) whereas the novelty in *FII* fell squarely *within* the legislative meaning (and so the question in *FII* was whether to *narrow* the legislation to exclude it). But this led them (wrongly) to doubt whether ‘the “always speaking” principle’ is relevant, although they concluded that it ‘ultimately does not matter’ because it ‘boils down to the same issue … what is the construction of the provision which best gives effect to the policy of the statute’?^100^ That Lords Reed and Hodge did not recognise that this type of ‘always speaking’ principle (ie a response to the novelty issue) governed *FII* may partly explain why they did not set out—or purport to apply—Lord Wilberforce’s tests from *Royal College of Nursing*.

The joint dissenting judgment of Lords Briggs and Sales did much better on this score. They considered it was ‘helpful and appropriate’ to view the case ‘through the prism of the doctrine that statutes are to be taken to be “always speaking”’; but then added, curiously, that ‘nothing really turns on this’.^101^ Lords Briggs and Sales then opined that the ‘always speaking’ principle concerns ‘whether Parliament can be taken to have intended by [legislation] passed at one point in time, using language directed to the circumstances at that time, to cover a new set of circumstances which has come into existence since then’—ie they clearly took the ‘always speaking’ principle to be a response to the *novelty* issue—and they set out Lord Wilberforce’s tests from *Royal College of Nursing*.^102^ But Lords Briggs and Sales then presented their application of those tests as an *interpretation* of the Act—‘the word “mistake” cannot, on a purposive construction, be construed to apply to’ mistake of law claims—when, clearly, this was a *recasting* of the legislation (treating it as if it were worded differently) to generate what they took to be a better outcome for the novelty.^103^

For Lords Hamblen and Burrows, the decision of the majority in *FII* ‘can be viewed as an application of the always speaking doctrine’ as it held that ‘the best interpretation of the Act should apply the purpose of the provision to the present, not the past, state of the law’.^104^ It is not fully clear what Lords Hamblen and Burrows meant by this, but it seems to treat the ‘always speaking’ principle as a principle of dynamic interpretation rather than as a response to the novelty issue. However, *FII* was not resolved by giving the Act its current meaning: the legislation just said ‘mistake’, and mistakes of law have been ‘mistakes’ throughout.

This concluded their review of the five ‘leading cases on the always speaking principle’, two of which—*Burstow* and *Owens*—were resolved by giving the legislation its current meaning whereas the other three—*Royal College of Nursing*, *Quintavalle* and *FII GLO—*were resolved by recasting the legislation (so that it deals with a novelty in a way that the judges considered to be more sensible).

### C. *The Decision in* News Corp

Lords Hamblen and Burrows held that, although ‘the always speaking principle is at the heart of’ News Corp’s appeal, it is ‘significantly limited’ by EU law considerations.^105^ They then applied Lord Wilberforce’s tests from *Royal College of Nursing*, and concluded that the ‘EU law constraints mean that this is a case in which … the always speaking principle falls to be applied at the less liberal end of the scale’ and that this, together with key differences between printed newspapers and their digital editions, made it ‘clear’ that they were not within ‘the same genus of facts’.^106^ This was the right decision, reached (ultimately) for the right reasons. But their definition of the ‘always speaking’ principle, and their review of what they took to be the five ‘leading cases’ on it, shows that they did not recognise the different issues at stake (and, especially, did not distinguish the meaning and novelty issues). They also declined to endorse Lord Leggatt’s judgment, which is more attuned to these points (and to which I now turn).

## 8. News Corp: *Lord Leggatt’s Concurrence*

Lord Leggatt wrote a concurring judgment in *News Corp* because, although he agreed with Lords Hamblen and Burrows as to the result, he considered that ‘important questions of principle are raised—in particular, regarding the so-called “always speaking doctrine” in interpreting statutes—on which my reasoning does not coincide in all respects with theirs'.^107^ And Lord Leggatt was fully justified in doing so, as his concurring judgment contains the most valuable judicial exposition of the ‘always speaking’ notion to date.

Lord Leggatt traced the ‘always speaking’ idea back to Coode’s work on present-tense drafting, and noted that it ‘seems ironic’ that it has ‘itself changed its meaning and come to be used to express a different idea from the one originally expounded by Coode’.^108^ Lord Leggatt found it ‘unclear how the description of legislation as “always speaking” acquired a sense among English lawyers so different from that in which it was originally used’, though he added that it may partly ‘reflect the influence of *Bennion on Statutory Interpretation*’.^109^ But he implicitly conceded that we are stuck with this mistake, and then sought to limit the phrase ‘always speaking’ to what he called—apparently interchangeably—‘ambulatory’, ‘dynamic’ or ‘evolutionary’ interpretation (ie he sought to limit the phrase to labelling a response to the *meaning* issue).^110^ Lord Leggatt reinforced this by objecting to uses of ‘always speaking’ to label a response to the *novelty* issue:

A case sometimes cited as an authority on the application of the ‘always speaking principle’ to technological development is *Royal College of Nursing* … A passage from the speech of Lord Wilberforce, who dissented, was quoted with approval in the *Quintavalle* case and is quoted by Lord Hamblen and Lord Burrows … That passage seems to me to be a helpful statement of how a court should approach any case where it is necessary to decide whether statutory language applies to a set of facts which its authors did not actually have (or are unlikely to have had or could not have had, it matters not) in contemplation. I do not read it as articulating anything which can usefully be described as an ‘always speaking doctrine’.^111^

Here, Lord Leggatt was influenced by the fact that, in *Royal College of Nursing*, the Lords had reached their conclusion ‘without any mention of any “always speaking principle”’.^112^ But this is anachronistic: as noted in section 7B(i), the phrase ‘always speaking’ did not appear in any reported UK case until 1997 and, although Rupert Cross had made a passing reference to ‘the somewhat quaint statement that a statute is “always speaking”’ in 1976, it was not used in the UK to label a response to the novelty issue until Bennion’s Code emerged in 1984 (ie three years after the Lords decided the *Royal College of Nursing* case).^113^

Lord Leggatt also commented on how his version of the ‘always speaking’ principle—ie as a principle of dynamic interpretation—might apply to four different types of change: ‘linguistic changes’, ‘changes in social attitudes and values’, ‘advance[s] in scientific understanding’ and ‘technological change’.^114^ These comments, although interesting, go awry in some respects (which, unfortunately, space prevents me from analysing here).^115^ The key points, for present purposes, are that Lord Leggatt distinguished three types of principle to which the ‘always speaking’ label is applied, noted that they respond to different issues (in our terms, the operation, meaning and novelty issues) and then acknowledged that it is important to disentangle these issues (as well as their associated principles).

## 9. Conclusion

Lord Leggatt’s analysis is an important step towards clear thinking on these crucial points of statutory interpretation and application. In this article, I have shown how to complete that journey: by carefully distinguishing the four types of principle that have come to be associated with the ‘always speaking’ notion—and the four distinct issues that those principles respond to—and by showing how they differ, and why it is important to disentangle them.

Others have noted that our use of the ‘always speaking’ label has become problematic. For Ian Loveland, ‘the “always speaking” formula is not a particularly attractive way of framing’ a principle of dynamic interpretation; Jeffrey Goldsworthy recently called it ‘too ambiguous, and potentially sweeping, to be useful’; and even Lord Steyn has recognised that there ‘are at least two strands to this principle’.^116^ Chad Jacobi recently wrote that:

Notwithstanding that its origin rests in explaining a style of drafting, the expression ‘always speaking’ … has become synonymous with the ‘ambulatory approach’ to interpretation … It is clear that Coode was not seeking to say anything at all about an ambulatory approach … The reason for this transformation in the meaning of the expression is unclear. But [it] probably no longer matters.^117^

I have argued that this ‘transformation’ is only a part of the story, and it *does* matter that judges should use the ‘always speaking’ label correctly. Whether they will do so remains to be seen.

## Footnotes

1 Johan Steyn, ‘Deference: a Tangled Story’ [2005] PL 346, 347.

2
*Elgizouli v Secretary of State for the Home Department* [2020] UKSC 10; *Fowler v HMRC* [2020] UKSC 22; *Test Claimants in the FII GLO v HMRC* [2020] UKSC 47; *T W Logistics Ltd v Essex CC* [2021] UKSC 4; *General Dynamics United Kingdom Ltd v Libya* [2021] UKSC 22; *A-G v Crosland* [2021] UKSC 58.

3 Andrew Burrows, *Thinking About Statutes* (CUP 2018) 21–2; *News Corp UK & Ireland Ltd v HMRC* [2023] UKSC 7 [75]–[79].

4 I say ‘at least’ especially because the ‘always speaking’ metaphor also has recently been associated with an anti-lapse/desuetude principle: see David Dodd, *Statutory Interpretation in Ireland* (Tottel 2008) 85, 266.

5 See nn 43 and 62. See also Martin D Kelly, ‘“Always Speaking” Provisions: Legal Transplants Gone Awry’ (2024) <https://ssrn.com/abstract=4738797>; Martin D Kelly, ‘The Novelty Issue: Should Judges “Recast” Laws to Deal with New Developments?’ (2024) <https://ssrn.com/abstract=4739055>.

6 For a detailed history of the ‘always speaking’ metaphor, see Kelly, ‘“Always Speaking” Provisions’ (n 5).

7 George Coode, *On Legislative Expression* (William Benning and Co 1845) 66.

8 My wording.

9 Coode (n 7) 63.

10 ibid 63, 66. This ‘mode of expression’ can be used ‘assuming the law to be always speaking’: ibid 66.

11 This explains why the phrase ‘always speaking’ is usually placed in single quotation marks (scare quotes). I first used this ‘No Exit’ example in Martin D Kelly, ‘Speaking Through Time’ (Legal-Phi, 4 January 2022) <https://legalphi.wordpress.com/2022/01/04/martin-kelly-speaking-through-time/>.

12 I previously called this the ‘application issue’ rather than the ‘operation issue’. But ‘operation’ is the better term, as an utterance/law may be operative without applying. I thank Angus Gunn KC for this suggestion.

13 In the UK, people who are closely related (by blood or marriage) are forbidden from marrying each other; very occasionally, Parliament overrides this by enacting a statute that grants them permission to marry.

14 Robert E Megarry, *Miscellany-at-Law: A Diversion for Lawyers and Others* (Wildy & Sons 2006) 345. Megarry noted that the ‘answer to this question remains as obscure as the source of the story’, and he also confessed that ‘the concluding portion about successors in title was a conscious gloss on my part’: ibid.

15
*R v Burstow; R v Ireland* [1998] AC 147 (my emphasis). Each of the other four Lords who heard these joined appeals explicitly endorsed Lord Steyn’s opinion (without further comment).

16 This point also applies to utterances that create a norm which forms part of a system of norms, such as the rules of games or sports. Hence I mention here speaking ‘systemically’ and not just speaking ‘legally’.

17 See eg Peter W Hanks, ‘The Content–Force Distinction’ (2007) 134 Philosophical Studies 141.

18 UK legislation can have legal effects even if it is not in force (eg it may prohibit conflicting Government actions): *R v Secretary of State for the Home Department, ex p Fire Brigades Union* [1995] 2 AC 513.

19 Theft Act 1968, ss 22 and 35.

20 This point also arises in other (non-legislative) legal contexts, eg in succession law. A will (executed at t_1_) only legally ‘speaks’ at a future date (t_2_) when the testator dies. The will creates ongoing duties from t_2_—mainly for the executors—some of which may be of indefinite duration (eg if the will creates trusts, especially if there is no rule against perpetuities). The operative provisions of wills are often not momentary, and some may be ‘always speaking’, but (at least under English law) they are operative only from the testator’s death. They create ‘virtually no legitimate third-party reliance interests’ until then: Martin D Kelly, ‘Mixed-up Wills, Rectification and Interpretation: *Marley v Rawlings*’ (2017) 38 Stat LR 265, 282; but see Alexandra Braun, *Claiming a Promised Inheritance: A Comparative Study* (OUP 2022).

21 Based on Coode’s approach, several jurisdictions (starting in Canada in the 1840s) have enacted ‘always speaking’ provisions—stipulating that their legislation is presumed to be ‘always speaking’—and these were generally understood to concern the *operation* issue: see Kelly, ‘“Always Speaking” Provisions’ (n 5).

22 On the key questions of (i) what, exactly, is the *original* time; (ii) what, exactly, is the *current* time; and (iii) whether these are the only options, see Martin D Kelly, ‘The Loquacious Legislature: Are Statutes “Always Speaking”?’ (PhD thesis, University of Edinburgh 2021) <https://ssrn.com/abstract=4738764> 14–22.

23 See eg *R (ZYN) v Walsall Metropolitan BC* [2014] EWHC 1918 (Admin) [39] (Leggatt J); Antonin Scalia and Bryan A Garner, *Reading Law* (Thomson/West 2012) 78; Ruth Sullivan, *The Construction of Statutes* (7th edn, LexisNexis Canada 2022) 169.

24 John F Burrows, ‘The Problem of Time in Statutory Interpretation’ [1978] NZLJ 253; William N Eskridge Jr, ‘Dynamic Statutory Interpretation’ (1987) 135 U Pa L Rev 1479; *Edwards v A-G* [1930] AC 124, 136.

25
*Gammans v Ekins* [1950] 2 KB 328; *Dyson Holdings Ltd v Fox* [1976] QB 503 (this was not decided until 1975, but the tenant had died in 1961); *Fitzpatrick v Sterling Housing Association Ltd* [2001] 1 AC 27.

26 Scalia and Garner (n 23) 78.

27 We might, of course, be justified in assuming that this *remained* Queen Anne’s opinion, unless and until we have evidence to the contrary. But that does not involve her utterance *operating* on an ongoing basis.

28 For the reasons why this qualifies as a change of *meaning*—specifically, as a change of *content* (understood as a function from facts to applications)—see Kelly, ‘The Loquacious Legislature’ (n 22) 38–58.

29 I first disentangled the operation and meaning issues in ibid 194–201. There, I called the latter the ‘temporal issue’; but there is a sense in which all of these issues are ‘temporal’.

30 Here I ignore that there may be a time gap between making the utterance and it coming into force (see s 6).

31 That is, it would commit the logical fallacy of converting a conditional. The conditional claim here is that ‘an utterance may change in meaning over time only if it is not momentary’, and this is not logically equivalent to its converse: ‘if an utterance is not momentary then it may change in meaning over time’.

32 This explains why, although many common-law jurisdictions have enacted an ‘always speaking’ provision, others—including Scotland, England & Wales, Australia (at federal level and in all but three states) and the United States (at federal and state level)—have never done so: see Kelly, ‘“Always Speaking” Provisions’ (n 5).

33 Canada, for example, has long endorsed an ‘original meaning rule’ for its federal legislation: see Elmer A Driedger, *The Construction of Statutes* (1st edn, Butterworths 1974) 134–5; Sullivan (n 23) 169–90. Some claim (wrongly) that this was historically the UK position: see Kelly, ‘The Loquacious Legislature’ (n 22) 160–2.

34 This oversimplifies matters. See Kelly, ‘The Loquacious Legislature’ (n 22) 201–30 for the fuller position.

35 My use of the word ‘novelty’ here is partly stipulative, as it may not align exactly with its ordinary usages. In particular, a novelty (in my sense) need not be something that did not exist, or that was unknowable, at the time of utterance. What matters here is that the producer was not aware of it when they chose their words.

36 We could, of course, just discard this advice. But there are other contexts (including, importantly, *legal* contexts) in which we have good reasons to follow an instruction even if it is now worded sub-optimally.

37 I borrow the term ‘recast’ from Lord Neuberger, who used it to describe the duty imposed by the Human Rights Act 1998, s 3: ‘If legislation does not appear to comply [with Convention rights], we must, if we can, recast it so that it does comply’: David Neuberger, ‘“Judge Not, That Ye Be Not Judged”: Judging Judicial Decision-Making’ (FA Mann Lecture, 2015) <www.supremecourt.uk/docs/speech-150129.pdf> accessed 16 March 2024 [47]. See also Aileen Kavanagh, *The Collaborative Constitution* (CUP 2023) 234. I previously called this ‘revising’—see Kelly, ‘The Loquacious Legislature’ (n 22) 26—but ‘recasting’ highlights the connection to the very similar action that judges perform when they apply s 3 of the Human Rights Act 1998.

38 Novelties may include inventions, new technologies and new types of situation (the latter including advances in our understanding). For more details (and many examples), see Kelly, ‘The Novelty Issue’ (n 5).

39 For a recent example, see Adrian Vermeule, *Common Good Constitutionalism* (Polity Press 2022) 96. Despite this lack of controversy, it is sometimes positively asserted that legislation is capable of applying to novelties: Peter B Maxwell, *On the Interpretation of Statutes* (2nd edn, W Maxwell & Sons 1883) 93; Glanville Williams, *Salmond on Jurisprudence* (11th edn, Sweet & Maxwell 1957) 153; Driedger (n 33) 134; Dennis C Pearce, *Statutory Interpretation in Australia* (1st edn, Butterworths 1974) 27; Pierre A Coté, *The Interpretation of Legislation in Canada* (1st edn, Les Éditions Yvon Blais 1984) 203–4; John Bell and George Engle, *Cross: Statutory Interpretation* (2nd edn, OUP 1987) 50; Andrew Burrows (n 3) 22.

40
*Aubrey v The Queen* [2017] HCA 18 [29] (joint opinion of Kiefel CJ and Keane, Nettle and Edelman JJ).

41
*Victor Chandler International Ltd v HMCE* [1999] 1 WLR 2160; Betting and Gaming Act 1981, s 9(1)(b).

42 Legislatures themselves may play a role in changing linguistic meaning. For example, *Keane v An Bord Pleanála* [1997] 1 IR 184 concerned whether a radar mast was a ‘beacon’ for the purposes of an 1894 Act. Barrington J claimed that ‘the dictionary definition of “beacon” was influenced by’ a UK Act of 1979 which stipulated that ‘beacon’ (as used in the 1894 Act) ‘shall be construed as including’ specified navigation aids; and that, partly because of this 1979 Act, we came to use the word ‘beacon’ in a broader sense: ibid 234.

43 Contrast *Fitzpatrick* (n 25), where a novelty—changed public attitudes to homosexuality—had become reflected in our use of ‘family’, such that the current meaning generated a more satisfactory outcome, with a Canadian case in which the novelty still fell outwith even the current (very different) meaning of that term. The latter concerned whether legislation (first enacted in 1908) which provided that a ‘juvenile delinquent’ could be ‘placed in a suitable *family* home as a foster home’ (my emphasis); the Supreme Court of Canada held that, despite the considerable changes in our use of the word ‘family’ since 1908, its meaning did not cover the novelty (a commercially operated ‘group home’ run by professional staff who all lived offsite): *Ontario (A-G) v Peel (Regional Municipality)* [1979] SCJ No 90. I am grateful to Chloë Kennedy for rightly pointing out that changed public attitudes may count as a ‘novelty’ (under my usage of that term).

44
*Fitzpatrick* (n 25). Lord Hutton, dissenting, assumed that ‘family’ here must be given its original meaning and concluded there was no novelty, as cohabiting homosexual couples were known to the 1920 Parliament: ibid 63. But, even if that is right, our (radically) changed social attitudes towards them *do* count as a novelty.

45 Offences Against the Person Act 1861, ss 20, 47; *Burstow* (n 15).

46
*Burstow* (n 15) 158–9.

47 I first distinguished the meaning and novelty issues in Kelly, ‘The Loquacious Legislature’ (n 22) 25–32.

48 Abortion Act 1967, s 1(1); *Royal College of Nursing of the UK v Department for Health & Social Security* [1981] AC 800. The full statutory phrase is ‘terminated by a registered medical practitioner’.

49
*Royal College of Nursing* (n 48) 806–14. Lord Denning’s suggested recasting (which he ultimately did *not* adopt) was to read the provision as if it contained the words ‘or by a suitably qualified person in accordance with the written instructions of a [doctor]’: ibid 807.

50 ibid 823, 831 (Lord Edmund-Davies). Such recasting has come to be called ‘updating’ (and adopting an ‘updating construction’)—terms which were coined by Bennion in his highly influential practitioner work: Francis AR Bennion, *Statutory Interpretation: A Code* (1st edn, Butterworths 1984) 355. But Bennion’s terminology here is perniciously ambiguous: for the reasons why, see Kelly, ‘The Novelty Issue’ (n 5).

51 Dynamic interpretation can, of course, also be justified on grounds other than parliamentary sovereignty (eg on moral grounds). But the claim that a judicial power to strain or recast legislation may be justified on sovereignty grounds—that Parliament intended that its legislation be strained or recast (in appropriate cases)—is considerably more problematic, for reasons I explore in some detail in Kelly, ‘The Novelty Issue’ (n 5). Here, my main goal is simply to show that the meaning and novelty issues are different; and that is surely true even if one thinks that responses to them may be justified more similarly than I have suggested here.

52 For the reasons why, see Kelly, ‘The Loquacious Legislature’ (n 22) 150–3.

53
*Stock v Frank Jones (Tipton) Ltd* [1978] 1 WLR 231, 237.

54 Evelyn Ellis, ‘Sources of Law and the Hierarchy of Norms’ in David Feldman (ed), *English Public Law* (OUP 2009) 72.

55 On the circumstances in which UK judges may be permitted—exceptionally—to limit the retrospective effect of a court decision, see *Re Spectrum Plus Ltd* [2005] 2 AC 680.

56 Jack Tsen-Ta Lee, ‘The Text Through Time’ (2010) 33 Stat LR 217, 235; Neil Duxbury, *Elements of Legislation* (CUP 2013) 229. For a detailed discussion (and critique) of Richard Ekins’s objections to judicial powers to recast (or, in his terms, ‘update’) legislation, see Kelly, ‘The Loquacious Legislature’ (n 22) 292–4. My view is that judges *should* have such a power (subject to certain conditions): see Kelly, ‘The Novelty Issue’ (n 5).

57 Another clear case is *R (Quintavalle) v Secretary of State for Health* [2003] 2 AC 687: see s 7B(iii) below.

58 This is subject to any authorised delegation of that power (eg through a so-called ‘Henry VIII clause’).

59 This is how the ‘doctrine of implied repeal’ is usually justified: ‘where the provisions of two statutes cannot stand together, the later provisions prevail and the earlier provisions are treated as repealed by implication or amended to the extent necessary to remove the inconsistency’: *Hamnett v Essex CC* [2017] EWCA Civ 6 [26] (Gross LJ). But implicit modifications are by no means limited to implied repeals.

60
*Oxfordshire County Council v Oxford City Council* [2006] UKHL 25; Commons Registration Act 1965, s 22. Beaches, car parks and urban wasteland may count as a ‘town or village green’ under this definition.

61 I first drew this distinction—and made these points—in Kelly, ‘The Loquacious Legislature’ (n 22) 33–7. There, I called this the ‘implicit *amendment* issue’; but it naturally arises for repeals as well as amendments.

62 Examples include *Oxfordshire* (n 60) [107] (Lord Scott) and *Moorthy v HMRC* [2018] EWCA Civ 847 [69] (Henderson LJ). *Moorthy* concerned whether a 1960 tax exemption for compensation to employees for an ‘injury’ was implicitly modified by 1975 legislation which granted employees a right to compensation ‘for injury to feelings’. Both cited *Victor Chandler* (n 41) in linking the decision to the ‘always speaking’ notion.

63 This reflects what I call the ‘Fundamental Rule of Utterance Interpretation’: language + context = content. Hence, the content expressed by an ‘always speaking’ utterance may change through the passage of time—even though the language (including the linguistic meaning of the words) used to express it stays the same—because the context has changed. See Kelly, ‘Speaking Through Time’ (n 11); Martin D Kelly, ‘Uncommunicative Legislating’ (2024) <https://ssrn.com/abstract=4740563>.

64 Necessary conditions are abundant: continuing to breathe is a necessary condition of achieving promotion, but it would be absurd to call a principle that we should continue to breathe ‘the promotion principle’.

65
*News Corp* (n 3) [2]. Lords Hamblen and Burrows delivered a joint judgment, with which two of the other Justices (Lords Hodge and Kitchin) agreed without further comment.

66 ibid [4].

67 ibid [29].

68 Andrew Burrows (n 3) 21 (footnote omitted) (my emphasis).

69
*News Corp* (n 3) [30].

70 ibid [30].

71
*Royal College of Nursing* (n 48) 822 (my emphasis).

72 Lord Wilberforce framed his test in terms of ‘extension’, no doubt because the question in *this* case was whether to extend the abortion legislation to cover medical inductions. But novelty issue cases can involve narrowing—rather than extending—legislation; an example is *FII GLO* (n 2), which I discuss in s 7B(v) below.

73
*Royal College of Nursing* (n 48) 822–3.

74 ibid 822.

75 ibid.

76
*News Corp* (n 3) [49].

77
*R v Hammersmith and Fulham LBC, ex p M* (1998) 30 HLR 10 [17] (Woolf MR).

78
*News Corp* (n 3) [32].

79
*Burstow* (n 15) 158. He traced it back only as far as Henry Thring, *Practical Legislation* (2nd edn, HMSO 1902) 83.

80
*Burstow* (n 15) 158.

81 ibid 158–9; see s 7B(i) above.

82
*Quintavalle* (n 57); Human Fertilisation and Embryology Act 1990, s 1(1).

83
*R (Quintavalle) v Secretary of State for Health* [2002] QB 628, 641 (Lord Phillips MR).

84
*Quintavalle* (n 57) [9].

85 For a fuller analysis of this *dictum*, see Kelly, ‘The Loquacious Legislature’ (n 22) 220–1.

86 ibid [9]–[10], [15].

87 ibid [15]–[16]. Despite the House of Lords recasting the 1990 Act in this way, Parliament eventually amended it to remove the reference to ‘fertilisation’: Human Fertilisation and Embryology Act 2008, s 1(2).

88
*Quintavalle* (n 57) [24].

89 ibid [26].

90 ibid. Lords Hamblen and Burrows said, somewhat misleadingly, that in *Quintavalle* ‘The words “where fertilisation is complete” were interpreted as laying down the time at which an embryo should be treated as an embryo rather than being integral to the definition of an embryo’: *News Corp* (n 3) [34]. Lord Millett did say this: *Quintavalle* (n 57) [46]. But none of the other Lords agreed, and his argument for it is weak: even if the ‘fertilisation’ wording relates only to time (a highly strained reading), this would not help, as CNR embryos are never fertilised, so they never reach the time when the Act would start to treat them as an ‘embryo’.

91
*Quintavalle* (n 57) [26].

92 ibid [22]–[23].

93 ibid [23]. Lord Steyn also said, curiously, that the fact that the legislation is ‘always speaking’ makes it ‘possible to consider whether the new technique … is covered by the Parliamentary intent’: ibid. This might suggest that he also took the notion that legislation is ‘always speaking’ to mean that it has ongoing *operation* (which, as we saw in s 6, is characteristically a precondition for the novelty issue to arise).

94
*Owens v Owens* [2018] UKSC 41; Divorce Reform Act 1969, s 2(1)(b).

95
*Priday v Priday* [1970] 3 All ER 554, 557; *Owens* (n 94) [34].

96
*Owens v Owens* [2017] EWCA Civ 182 [41].

97 Limitation Act 1939, s 26(c); *Kleinwort Benson Ltd v Lincoln CC* [1999] 2 AC 349. The Limitation Act allowed recovery within six years of when the mistake was discovered (or, if earlier, it became reasonably discoverable).

98
*FII GLO* (n 2) [218] (Lords Reed and Hodge presumably used the word ‘connotation’ here as a synonym for ‘meaning’); *Fitzpatrick* (n 25) (s 4 above); *Burstow* (n 15) (ss 4, 7B(ii) above).

99
*FII GLO* (n 2); *Royal College of Nursing* (n 48) (s 7B(i) above); *Victor Chandler* (n 41); *Quintavalle* (n 57) (s 7B(iii) above).

100
*FII GLO* (n 2) [218]–[219].

101 ibid [268].

102 ibid [269]–[270]. Lords Briggs and Sales correctly understood the first part of that test, and they concluded that the novelty (actions in restitution for mistake of law) did not ‘fall within the same genus’ as actions in restitution for mistakes of fact: ibid [274]. But they did not adjust the second part of the test for a case of narrowing (rather than broadening) the legislation, leading them to conclude that there was no ‘clear purpose in the legislation which can only be fulfilled if’ the novelty is included: ibid. This was not the right question: the novelty already fell within the legislative meaning, and the correct question was whether there was a clear legislative purpose that required *excluding* actions for mistake of law from the special limitation period.

103 ibid. Recall that the legislative wording was ‘action … for relief from the consequences of a mistake’. A mistake of law is just as much a 'mistake' as a mistake of fact.

104
*News Corp* (n 3) [37]. Lords Hamblen and Burrows did not comment on the distinction drawn by the majority in *FII* (Lords Reed and Hodge) between, in our terms, the meaning issue and the novelty issue.

105 ibid [48].

106 ibid [49]–[58]. VAT law is harmonised across the EU, and zero-rating was a derogation that the UK negotiated on its accession to the EEC. By ‘standstill provision’,they meant that ‘the categories of zero-rating cannot be expanded or extended beyond those which existed on 31 December 1975’: ibid [40]. On the VAT aspects of the *News Corp* case, see Max Schofield, ‘*News Corp UK and Ireland Ltd v HMRC*: Always Speaking in the Supreme Court—a Strictly Neutral Approach’ [2024] BTR 52.

107
*News Corp* (n 3) [65].

108 ibid [75]–[78].

109 ibid [79]. Lord Leggatt is right that Bennion is largely responsible for spreading this confusion across the common-law world: see Kelly, ‘“Always Speaking” Provisions’ (n 5); Kelly, ‘The Novelty Issue’ (n 5).

110
*News Corp* (n 3) [79]. He (rightly) cited *Burstow* as authority for this, but also (wrongly) cited *Quintavalle*.

111 ibid [93].

112 ibid.

113 Rupert Cross, *Statutory Interpretation* (1st edn, Butterworths 1976) 45; Bennion (n 50).

114 ibid [82]–[93]. Lord Leggatt recognised that this was not ‘a full analysis’: ibid [81].

115 For a more detailed analysis of these comments of Lord Leggatt, see Kelly, ‘The Novelty Issue’ (n 5).

116 Ian Loveland, ‘Freedom of Political Expression: Who Needs the Human Rights Act?’ [2001] PL 233, 241 (Loveland favours the label ‘evolving purpose doctrine’ for this); Jeffrey Goldsworthy, ‘Lord Burrows on Legislative Intention, Statutory Purpose, and the “Always Speaking” Principle’ (2022) 43 Stat LR 79, 101; Johan Steyn, ‘The Intractable Problem of the Interpretation of Legal Texts’ (2003) 25 Syd LR 5, 12.

117 Chad Jacobi, *Interpretation Acts: Origins and Meaning* (Thomson Reuters 2019) 132–3.

## Notes

For their valuable comments on earlier drafts, I thank Deval Desai, Başak Etkin, Jeffrey Goldsworthy, Angus Gunn KC, Chloë Kennedy, Lucas Miotto, Thomas Poole, Paul Roberts, Paolo Sandro, Adam Tomkins and the anonymous reviewers.

